# An Interactive Constraint-Based Decision-Support Tool for Pharmaceutical Formulation Development

**DOI:** 10.3390/pharmaceutics18060635

**Published:** 2026-05-22

**Authors:** Reihaneh Manteghi, Eduardo Veas

**Affiliations:** 1 Institute of Human-Centred Computing, Graz University of Technology, Sandgasse 36, 3rd Floor, 8010 Graz, Austria; eveas@tugraz.at; 2 Know Center Research GmbH, Sandgasse 34, 2nd Floor, 8010 Graz, Austria

**Keywords:** pharmaceutical formulation, decision-support systems, constraint satisfaction problem, interactive visualization, data-driven formulation

## Abstract

**Background/Objectives:** Pharmaceutical formulation involves designing a drug product by combining the properties of an active pharmaceutical ingredient (API) with suitable excipients and processing strategies to produce a safe, effective, and manufacturable dosage form. However, data in formulation science are often limited, expensive to generate, and frequently restricted by proprietary and confidentiality constraints. Interactive digital tools can support formulators during early drug product development by improving the structure, transparency, and efficiency of formulation decision-making. While the current system focuses on structured decision support, future extensions may incorporate machine-learning methods for recommendation and knowledge extraction. **Methods:** In this work, we developed the Formulation tool, an interactive decision-support and visualization system for formulation development based on a hierarchical formulation-strategy framework commonly used in pharmaceutical practice. The tool is designed to prioritize suitable formulation principles and associated processing routes, with oral solid formulation as the initial application domain. The evaluated scenarios also include pathway regions relevant to oral liquid formulations. Its modular architecture also makes it adaptable to other formulation scenarios. To assess practical applicability, the tool was evaluated in a structured expert study involving five expert users across six predefined formulation scenarios (*n* = 30 runs), covering three drugs under adult and pediatric conditions. **Results:** The tool showed agreement with the expected dosage-form families and overall formulation properties, with adult scenarios converging to oral solid regions and pediatric scenarios converging to oral liquid regions. At the downstream formulation-profile level, users converged either to the dominant expected pathway or to alternative feasible pathways within the same formulation region. Variability in full pathway completion was observed and was primarily associated with differences in user interaction behavior and exploratory usage patterns. The median task completion time was 113.5 s. **Conclusions:** In addition to organizing formulation knowledge, the Formulation tool records user interactions in a structured manner, which may support future learning from usage patterns. Because detailed downstream formulation constraints are often institution-specific and are typically not available in the public domain, the present evaluation focused on agreement at the dosage-form-family level and on overall formulation properties rather than on highly specialized constraint logic. The system is based on a constraint satisfaction problem (CSP) framework, which is well suited for modeling complex decision processes under explicit constraints. CSP has also been widely applied in intelligent scheduling systems, supporting its suitability for structured, constraint-rich decision-making tasks such as pharmaceutical formulation strategy development.

## 1. Introduction

Oral formulation development is becoming increasingly complex due to multiple challenges, ranging from costly experimentation to limited availability of high-quality data. To address these challenges, pharmaceutical development teams must adopt structured and systematic approaches to the design of formulations and manufacturing processes. Pharmaceutical formulations must meet multiple criteria, including safety, efficacy, manufacturability, and regulatory compliance, throughout development and in the final product. The complex physicochemical properties of modern drug candidates, combined with increasing pressure on development timelines and resource efficiency, have motivated the development of structured decision-support approaches for formulation design. These approaches aim to guide the selection of dosage forms, formulation strategies, and processing routes in a transparent and reproducible manner.

Meanwhile, the amount of digital data in the pharmaceutical industry is steadily increasing, and machine-learning methods are becoming more common. For instance, Lou et al. review how machine learning has been applied in solid oral dosage-form development over several decades [1]. Vora et al. provide a broad overview of AI in pharmaceutical technology, including formulation, testing, and delivery systems [2]. Despite important advances in data-driven methods in fields such as drug discovery [3], there is still substantial unexplored potential for leveraging data science in pharmaceutical research beyond discovery and manufacturing [4,5,6]. Formulation science, which is the focus of this work, often relies on relatively small datasets [6]. As a result, much of the existing data-science and machine-learning research in formulation has focused on specific prediction tasks, such as release optimization and release prediction.

In recent years, artificial intelligence, particularly artificial neural networks (ANNs), has been increasingly applied to the optimization of pharmaceutical formulations. ANNs have been successfully applied to evaluate and predict release profiles in modified-release solid dosage forms [7,8], optimize dissolution behavior in sustained-release systems [9,10], and even tailor release properties in innovative manufacturing techniques such as 3D printing [11]. These studies demonstrate the capability of AI models to capture complex, nonlinear relationships between formulation and process parameters that are difficult to express through traditional design-of-experiments methods. Beyond release modeling, AI and process analytical technologies are increasingly supporting real-time release testing and quality assurance [12]. Historically, statistical optimization approaches based on design of experiments (DoE), including factorial designs, [13] laid the groundwork for these more advanced AI-based predictive models.

One important application area is solubility prediction. Solubility prediction plays a critical role in pharmaceutical formulation, as poor solubility remains a key challenge for oral dosage form development. Multiple studies highlight the evolution of solubility prediction from purely empirical experimentation to integrated computational modeling, forming a foundation for data-informed formulation design. Recent advances in computational modeling and machine learning have significantly enhanced our ability to predict solubility with improved accuracy and efficiency. Hybrid approaches combining physics-based simulations with data-driven methods have demonstrated synergistic potential for improving predictive reliability [14]. Machine learning techniques, including Random Forests, Support Vector Machines, and Neural Networks, have been successfully applied to predict intrinsic solubility of drug-like molecules, showcasing strong performance in both generalization and accuracy [15,16]. However, the quality and reproducibility of experimental solubility data remain a limiting factor, as demonstrated by multi-laboratory evaluations of intrinsic solubility measurement techniques [17].

Understanding the material properties of solid dosage formulations is also essential for ensuring product quality, manufacturability, and performance. Multiple studies illustrate the evolution of computational and AI-assisted methods for optimizing material properties in solid formulation design. Early studies applied chemometric approaches to streamline formulation design, as demonstrated by Lindberg and Gabrielsson, who used computational software to assist tablet formulation optimization [18]. With the advent of advanced data analytics and machine learning, more recent research has explored the relationship between formulation structure and mechanical properties. For instance, Lou et al. employed machine learning algorithms to analyze the effects of core/shell particle engineering on powder compactability, improving the understanding of material performance in tableting processes [19]. Similarly, Takagaki et al. developed a tablet formulation database and used ensemble artificial neural networks to predict key pharmaceutical characteristics such as hardness and disintegration time, highlighting the predictive power of AI in solid dosage form development [20]. More recent work by Hayashi et al. applied machine-learning models to predict tablet properties such as tensile strength and disintegration time from material attributes and compression conditions, while Ficzere et al. used machine vision and deep learning for real-time coating-thickness measurement and defect recognition in film-coated tablets, further illustrating the growing integration of artificial intelligence into formulation and pharmaceutical product development [21,22].

Additional applications of data-driven and computational methods in formulation science include particle-size distribution prediction [23], co-crystallization success-rate estimation [24], and process analytical technologies (PAT) or online process monitoring [25]. Despite this broad applicability, machine learning in formulation science remains focused mainly on isolated prediction tasks rather than on supporting structured decision-making processes.

At the same time, formulation science offers considerable potential for improved data management and secondary data use [6]. Formulation scientists increasingly seek practical digital tools that can support development workflows using both historical and newly generated data. Several commercial systems address selected aspects of this need. For example, proprietary software such as Formulator can support inventory, data, and recipe management, while the online “Formulation Tool” from DFE Pharma provides formulation advice based on inputs such as API type, particle size, dose, and compatibility [26].

Nevertheless, existing commercial tools typically address specific formulation tasks, and no general-purpose, constraint-based decision-support system for formulation strategy is currently available. Motivated by this gap, we aim to develop a general, flexible, and modular tool that supports formulators in managing data, improving data quality, recording user interactions, and providing a foundation for future decision support. In this work, we present a software concept to address these challenges in formulation science, with oral solid dosage forms as the initial application domain. Although the underlying framework architecture is general and can represent broader formulation pathways, the present manuscript is motivated primarily by oral solid formulation development, while the evaluated scenarios also include pathway regions relevant to oral liquid formulations. Other route-specific and non-pharmaceutical examples are included only to illustrate extensibility rather than comprehensive validated coverage.

The methodological framework presented in Section 3 is organized into four main components:

1. Formulation strategy;
2. Constraint satisfaction problem;
3. Overview of the tool;
4. Evaluation design.

**Figure 1:**
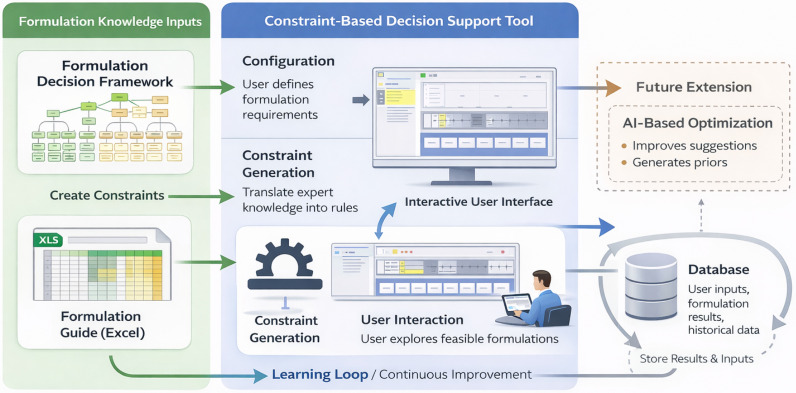
Process diagram of the proposed formulation decision-support framework. Expert formulation knowledge is structured as a formulation decision framework, representing constraint-based decision logic (e.g., decision-tree-like relationships), and complemented by formulation guides. This knowledge is translated into constraints within a digital decision-support tool that enables interactive exploration of feasible formulation pathways. User interactions and generated formulation results are stored to ensure traceability and support future system extensions. The integration of AI-based optimization methods is envisioned as a potential future enhancement of the framework.

The overall workflow of the proposed framework is shown in Figure 1.

## 2. Related Work

This section reviews prior research and tools related to drug formulation design and decision-support systems and briefly compares the Formulation tool with existing approaches in the field.

### 2.1. Overview of Related Work

Drug formulation development requires balancing multiple constraints related to physicochemical properties, manufacturability, stability, and in vivo performance. In response to these challenges, decision-support approaches for formulation and preformulation have been explored for decades, ranging from knowledge-based expert systems to modern data-driven methods.

Early work includes rule-based and knowledge-based systems intended to capture expert reasoning and standardize formulation decisions. For example, Ramani et al. described the design and implementation of an expert system to support drug preformulation activities in an industrial setting [27]. More formulation-specific expert systems have also been developed, such as ESFppop, a rule-based expert system designed to shorten development time for push–pull osmotic pump tablets of poorly water-soluble drugs by combining a rule base with models of release behavior and a formulation design module [28]. More recently, Wang et al. introduced PharmDE, an expert system that integrates database searching with rule-based incompatibility risk prediction to support drug excipient compatibility assessment and accelerate formulation design [29].

Alongside expert systems, data-driven approaches using statistical learning and machine learning (ML) have become increasingly common in formulation research. A comprehensive review by Lou et al. summarizes three decades of ML applications in solid oral dosage form development, spanning preformulation, formulation design, and process development tasks [1]. In parallel, newer digital decision-support platforms also demonstrate integration of heterogeneous data sources for practical decision-making. For instance, Grigoryan et al. report an AI-driven web platform that integrates molecular descriptors, formulation variables, and environmental parameters to support stability-related decision-making for compounded oral solid dosage forms [30].

Although these systems are valuable, they address different decision-support needs. PharmDE focuses on drug–excipient incompatibility assessment through database-supported and rule-based risk evaluation, and Smart Formulation focuses on predictive support for stability and beyond-use-date estimation in compounded oral solid dosage forms. By contrast, the present Formulation tool is designed as an interactive hierarchical decision framework for structured exploration of feasible formulation pathways across multiple formulation stages. Its distinguishing contribution lies in the combination of explicit rule-based knowledge representation, graph-based pathway navigation, real-time feasibility evaluation, and an administrative interface that allows domain experts to modify and maintain the encoded decision logic over time.

Prior work in formulation decision support has largely followed two complementary directions: knowledge-based systems that formalize expert rules and domain heuristics, and data-driven approaches that learn predictive relationships from experimental and curated datasets. Building on these foundations, the present work introduces a decision-support tool for drug formulation that represents the process as a structured decision tree augmented by constraint satisfaction. This approach enables systematic exploration of feasible formulation pathways while maintaining explicit representation of formulation constraints and preserving expert control over final decisions.

### 2.2. Previous Work on Decision Support in Drug Formulation

Previous research on decision-support systems in pharmaceutical development can be broadly categorized into (i) model-based decision-support frameworks, (ii) data-driven and artificial intelligence (AI) approaches across the drug-development lifecycle, and (iii) formulation-focused predictive modeling and decision support.

Model-based decision-support frameworks have been used to improve strategic and operational decision-making in drug development. Wiklund [31] proposed a quantitative modeling framework that integrates mechanistic models, computer simulations, and data-driven components to evaluate development options and support transparent decision-making. A key characteristic of this framework is its holistic view of the development program, combined with explicit handling of uncertainty through simulation-based evaluation. Although such approaches are not formulation-specific, they establish important principles for structured decision support, including data integration, interpretability, and flexibility across development stages.

In parallel, formulation-specific decision-support methods have been developed, particularly in the context of preformulation and material characterization. One representative example is the SeDeM expert system, which is primarily designed to assess the suitability of powders for direct compression in tablet formulation [32,33]. SeDeM relies on experimentally determined material parameters that are transformed into quantitative indices to guide formulation decisions, such as excipient selection and formulation feasibility for direct compression. While effective within its intended scope, this approach focuses on material-level assessment and does not explicitly model multi-stage formulation decision processes or dependencies between formulation steps.

Compared to these approaches, the proposed Formulation tool addresses a different aspect of formulation decision-making. Rather than relying on simulation-based evaluation as in model-driven frameworks or on parameter-based material assessment as in SeDeM, the present work focuses on structuring formulation reasoning through explicitly encoded rule-based constraints. The tool represents formulation knowledge as a hierarchical decision graph in which formulation options are evaluated based on logical compatibility between variables. This enables interactive exploration of feasible formulation pathways while maintaining full expert control over decision-making. A qualitative comparison of these approaches is provided in Table 1.

Importantly, the objective of the proposed approach is not to identify an optimal solution, but to determine the set of logically feasible formulation pathways and to prevent inconsistent combinations of formulation decisions. In this sense, the tool is complementary to both model-based frameworks and quantitative formulation methods, as it supports early-stage formulation reasoning where data may be limited and decisions rely heavily on expert knowledge.

In parallel, data-driven and AI-based methods have gained significant attention across the pharmaceutical lifecycle. Reviews such as that by Liang et al. [34] illustrate the use of AI in cancer drug development and precision therapy, including target identification, efficacy and toxicity prediction, dose optimization, and treatment personalization. Similarly, Mohanty et al. [35] describe AI-driven approaches for drug repurposing during the COVID-19 pandemic, emphasizing the importance of curated training datasets and appropriate learning algorithms to improve prediction efficiency. While these studies demonstrate the transformative potential of AI for decision-making in pharmaceutical research, their primary focus lies in drug discovery, repositioning, and clinical decision support rather than formulation-level decision processes.

More closely aligned with formulation science, machine learning has increasingly been applied to support formulation development tasks and to predict formulation-relevant properties. Lou et al. provide a comprehensive review of machine learning applications in solid oral dosage form development, covering formulation and process development problems where data-driven models can complement experimental work [1]. As an example of property-level prediction, Youshia et al. developed an artificial neural network model to predict particle size of polymeric nanoparticles from formulation and process parameters, demonstrating how data-driven models can capture nonlinear relationships in formulation systems [36].

Despite these advances, much of the existing literature focuses on predicting or optimizing isolated formulation attributes. There remains a need for interactive decision-support tools that formalize formulation strategy as a structured decision process, integrate expert knowledge with explicit constraints, and support traceable decision pathways during formulation development. Addressing this gap motivates the constraint-based, decision-tree approach proposed in this work.

## 3. Materials and Methods

This section describes the conceptual and methodological foundations of the proposed Formulation tool. The case study and experimental evaluation were conducted to demonstrate the applicability of the proposed decision-support framework in pharmaceutical formulation development.

**Figure 2:**
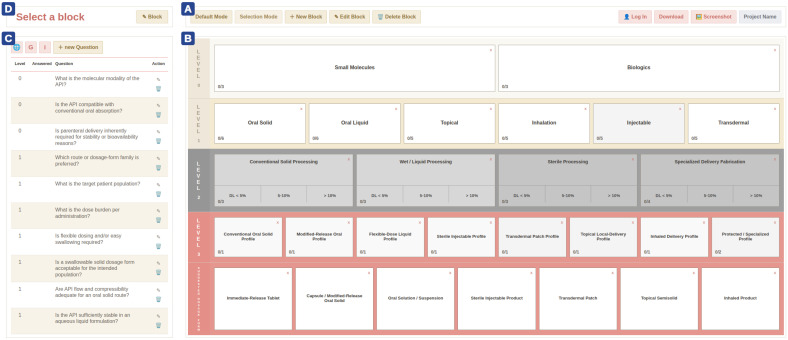
Overview of the formulation decision-support tool user interface, illustrating the formulation strategy as a graph-based question–answering workflow. (**A**) Control bar providing access to user management, process overview, graph structure management, and process mode control, enabling alternative workflows. (**B**) Decision graph representing the formulation strategy, including hierarchical decision levels, formulation blocks, and their interconnections. (**C**) Constraint table listing block-specific questions that define formulation constraints, with options to edit, remove, or add entries. (**D**) Block details panel displaying detailed information about the selected block, associated metadata, and options for manual configuration or document attachment.

The Materials and Methods are organized into four main subsections: Section 3.1 (Formulation Strategy), Section 3.2 (Constraint Satisfaction Problem), Section 3.3 (Overview of the Tool), and Section 3.4 (Evaluation Design). The Formulation Strategy subsection introduces the underlying formulation concept and outlines the decision logic used to prioritize formulation principles and processing routes during drug product development. The Constraint Satisfaction Problem subsection formalizes the formulation decision task as a configuration problem and describes the methods used to translate the formulation strategy into an interactive, constraint-driven exploration framework. The Overview of the Tool subsection presents the software implementation, highlighting the core features, functionalities, and user interactions supported by the system. Finally, the Evaluation Design subsection describes the structured expert study, predefined formulation tasks, and quantitative and qualitative metrics used to assess the practical applicability of the tool. An overview of the Formulation tool user interface is shown in Figure 2.

### 3.1. Formulation Strategy

The formulation strategy used in this work is designed to support systematic decision-making in the development of oral solid dosage forms by prioritizing suitable formulation principles and associated processing routes. The primary objective of this strategy is to establish a shared and structured understanding of critical Active Pharmaceutical Ingredient (API) properties and formulation requirements, thereby enabling the development of robust, efficient pharmaceutical manufacturing processes and optimal drug product performance for patients.

The formulation strategy prioritizes formulation options based on key API properties, project-specific constraints, and biopharmaceutical requirements that influence the intended in vivo performance of the drug product. To support this prioritization, the decision process is organized into multiple sequential phases, which are conveniently structured as a decision matrix. Each phase represents a distinct level of decision-making, guiding the formulator through progressively refined formulation choices.

This structured organization facilitates cross-project learning, supports controlled knowledge management, and accelerates formulation development by focusing experimental efforts on options with the highest probability of success. By reducing redundant experimentation and enabling early elimination of less suitable formulation pathways, the strategy allows technical development to concentrate on the most relevant experiments and supports earlier, more informed decision-making.

The formulation strategy is based on publicly available formulation development methodologies and commonly used formulation decision criteria reported in pharmaceutical development literature [2,18]. These resources serve as a reference framework for applying the strategy consistently across projects and form the basis for its translation into the interactive digital decision-support tool presented in this work.

### 3.2. Constraint Satisfaction Problem

Decision-making tasks are particularly challenging in complex, multi-stage processes that involve limited or evolving input data. In such settings, the formal design of the decision task plays a crucial role in supporting transparent, consistent, and traceable decision-making. Pharmaceutical formulation development exemplifies this challenge, as formulation scientists must navigate numerous interdependent decisions under technical, temporal, and economic constraints while retaining expert control over final choices.

The proposed formulation decision framework follows an expert-in-the-loop paradigm. Formulation scientists retain full control over all decisions, while the system supports the decision process by enforcing logical consistency between formulation parameters. Rather than automating decision-making, the framework guides users through a structured exploration of feasible formulation pathways by preventing incompatible combinations of choices.

Scheduling and configuration systems represent a well-established class of decision-support applications designed to assist users in exploring complex decision spaces. A foundational modeling paradigm underlying such systems is the Constraint Satisfaction Problem (CSP), which provides a formal framework for representing decision variables, their admissible domains, and the constraints governing allowable combinations of variable assignments [37].

In contrast to optimization-based formulations of CSP, the objective in the present work is not to identify an optimal solution, but to determine the set of feasible formulation pathways that satisfy the defined constraints. When multiple feasible pathways are identified, final selection is performed by the formulation expert based on external criteria such as manufacturability, cost, or project-specific requirements.

The formulation decision process is defined by a set of variables $V=\{v_{1},\ldots ,v_{n}\}$, each corresponding to a formulation-related question presented to the user. Each variable is associated with a domain $dom(v_{k})$ consisting of admissible answer options. These domains are primarily categorical, although range-based inputs can also be represented.

Variables are organized into a sequence of hierarchical decision levels $L=\{l_{1},\ldots ,l_{i}\}$, where each level contains a set of formulation blocks $B=\{b_{i,1},\ldots ,b_{i,j}\}$. Each block $b_{i,j}$ represents a formulation option or decision unit and is associated with a subset of variables $V_{i,j}\subseteq V$. Blocks are connected across levels, forming a directed graph that defines feasible transitions between formulation decisions.

**Figure 3:**
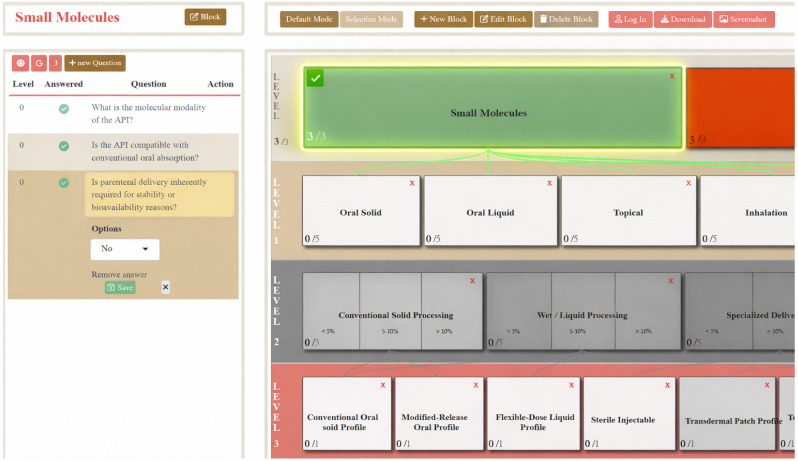
Interface representation of the formulation decision framework, illustrating the relationship between decision levels $L=\{l_{1},\ldots ,l_{j}\}$, formulation blocks $B=\{b_{i,1},\ldots ,b_{i,j}\}$, and variables $V=\{v_{1},\ldots ,v_{n}\}$. The figure highlights the currently selected formulation block in the decision graph (*Small Molecules*), the active associated question in the variable list (*Is parenteral delivery inherently required for stability or bioavailability reasons?*), and the recorded answer option. These highlighted elements demonstrate how variables associated with a selected block define constraint conditions and how user-provided answers are used to evaluate downstream formulation options, resulting in visible block validation states within the next decision level.

Figure 3 illustrates the relationship between decision levels, formulation blocks, and variables within the decision framework, highlighting the currently selected block and its active associated question.

Each block defines a corresponding set of constraint evaluations $C_{i,j}=\left\{c_{i,j,1},\ldots ,c_{i,j,k}\right\}$ derived from expert knowledge and user input. In the present implementation, constraints are explicitly encoded as rule-based mappings between user-provided answers and block validity states. As shown in Figure 3, selecting and answering a block-specific question not only records the relevant constraint condition but also directly affects the validation state of downstream candidate blocks in the decision graph.

Constraint evaluation is qualitative and rule-based. Each answer provided by the user assigns a validity state to one or more blocks according to the encoded formulation knowledge. These states are defined as:

- **Valid**: The answer is fully consistent with the formulation logic of the block.
- **Invalid**: The answer contradicts the formulation logic, and the block cannot be part of a feasible pathway.
- **Conditionally feasible**: The answer falls into an intermediate feasibility state between clearly valid and clearly invalid. In the underlying JSON representation of the tool, this intermediate state is labeled maybe_valid.

The *conditionally feasible* state, represented in the software logic as maybe_valid, reflects the inherent uncertainty in early-stage formulation development, where feasibility cannot always be determined conclusively at a given decision step. It is not a probabilistic score, but a rule-based intermediate validity category whose meaning depends on the specific constraint associated with each question. This state may arise either from quantitative threshold logic, when an input value falls between clearly valid and clearly invalid limits, or from qualitative expert-defined logic, when compatibility depends on additional downstream decisions or contextual information. This allows users to continue exploring potentially viable formulation routes while refining decisions iteratively.

A formulation pathway is considered feasible if, at each decision level, at least one connected block remains valid or conditionally feasible. Block validity is updated dynamically as user input evolves, allowing both forward exploration and backward revision of decisions.

To support expert interpretation, each block stores two indicators, $P_{\mathrm{valid}}\left(b_{i,j}\right)$ and $P_{\mathrm{invalid}}\left(b_{i,j}\right)$, representing the proportion of satisfied and unsatisfied constraint evaluations. These indicators are informative only and do not determine the final decision. In future versions of the tool, these weighted indicators will be leveraged by machine learning models that learn from user interactions and historical decisions to further enhance decision support.

All formulation knowledge and constraint definitions are stored in structured JSON files. During user interaction, selected answers and resulting block validity states are also recorded in JSON format, enabling reconstruction and visualization of the explored formulation pathway.

The following pseudocode summarizes the rule-based evaluation procedure used to determine block validity and identify feasible formulation pathways.

Algorithm 1 outlines the rule-based evaluation procedure used to determine block validity and identify feasible formulation pathways.   
**Algorithm 1:** Constraint-Based Evaluation of Formulation Pathways 
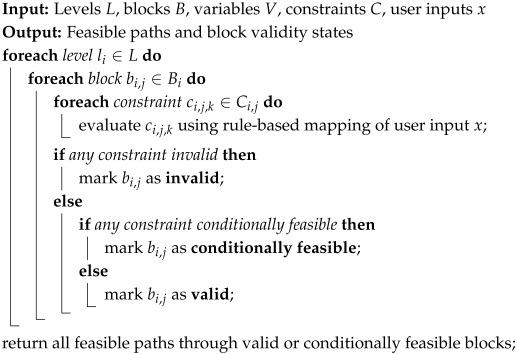


### 3.3. Overview of the Tool

**Figure 4:**
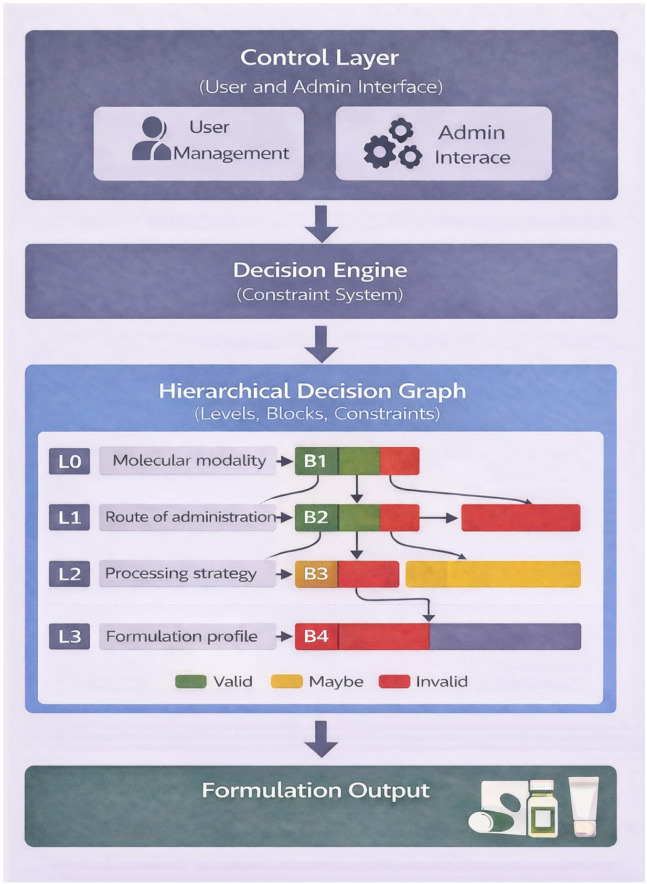
Conceptual architecture of the formulation decision-support framework. The system is organized into four layers: (i) a control layer enabling interaction through user and administrative interfaces, (ii) a constraint-based decision engine that evaluates formulation rules, (iii) a hierarchical decision graph representing formulation stages through levels and interconnected formulation blocks, and (iv) a formulation output layer that provides final dosage-form recommendations. Constraint evaluation dynamically propagates through the decision graph, updating block validity states and guiding users toward feasible formulation pathways.

The Formulation tool is an interactive, web-based decision-support application designed to assist formulation scientists in navigating complex formulation strategies through a structured, constraint-based configuration approach. The tool implements the formulation strategy described in Section 3.1 by translating its decision logic into an interactive exploration process grounded in constraint satisfaction principles, as detailed in Section 3.2. The overall conceptual architecture of the system is illustrated in Figure 4, highlighting the interaction between user interfaces, constraint evaluation, hierarchical decision structures, and formulation outputs.

The tool is designed to reduce cognitive complexity in formulation development by explicitly representing dependencies between formulation decisions. By structuring the decision process and highlighting relationships between parameters, the system assists users in avoiding inconsistent or incompatible combinations of formulation choices.

The underlying formulation knowledge is derived from hierarchical formulation development strategies commonly used in pharmaceutical research and is enriched with explicitly defined formulation constraints captured in accompanying spreadsheet-based resources. These constraints encode formulation-relevant requirements and decision criteria and form the knowledge base that drives the configuration logic of the tool. User inputs are evaluated against these constraints to dynamically determine feasible, conditionally feasible, and infeasible formulation pathways.

The current version of the Formulation tool focuses exclusively on interactive, constraint-based exploration of formulation strategies and does not include automated or artificial intelligence-based recommendation components. The tool is intentionally designed to support iterative, expert-driven decision-making rather than enforcing a single predefined workflow. Users may revisit and revise earlier decisions, explore alternative formulation pathways, and compare feasible options based on expert judgment and external considerations such as time, cost, or material availability.

In the following subsections, we describe (i) the tool design and user interface features, (ii) the development methodology and architectural considerations, and (iii) the core functional capabilities that support interactive formulation strategy exploration and decision support.

#### 3.3.1. Tool Design and Features

The overall structure of the Formulation tool is illustrated in Figure 1. The tool integrates a drug formulation strategy manual and explicitly encoded formulation constraints into an interactive, graph-based decision-support environment. The formulation strategy is represented as a graphical decision structure composed of hierarchical levels and interconnected formulation blocks, enabling users to explore formulation pathways and identify feasible formulation solutions through interactive constraint evaluation.

The tool is designed to support exploratory and iterative formulation decision-making. Users interact with the formulation strategy by navigating between hierarchical levels, formulation blocks, and associated questions (variables). By combining expert-defined formulation knowledge with user-provided inputs, the tool enables structured exploration of formulation options while maintaining transparency and traceability throughout the decision process.

##### Development Methodology

The Formulation tool is implemented as a web-based application using R Shiny (https://shiny.rstudio.com/ is an R package for interactive web app development) for backend logic and application flow, and D3.js (https://d3js.org/ is a JavaScript library for manipulating documents based on data) for interactive data visualization. This architecture enables dynamic rendering of graph-based decision structures and real-time interaction with formulation data. All formulation knowledge, including the definition of levels, blocks, variables, constraints, and their relationships, is stored in structured JSON files. This data-driven design allows the formulation strategy to be easily modified, extended, or replaced without changes to the core application logic. All graph elements, table components, block–question relationships, and constraint definitions are described within JSON objects. This modular representation supports flexibility, reuse across projects, and straightforward adaptation of the tool to alternative formulation strategies or future application domains. To support transparency and reproducibility, the public source code repository is available at https://github.com/reihanehmanteghi91/drug-formulation (accessed on 28 April 2026), and a live deployed version of the application is accessible at https://019db467-c018-09fc-19d0-e4edc7395e43.share.connect.posit.cloud/ (accessed on 25 April 2026).

##### Functional Capabilities

The primary functionality of the tool enables bidirectional exploration of the formulation strategy. Users may select a formulation block or hierarchical level to view the associated questions and constraints, or alternatively select a question (variable) to visualize all related formulation blocks. This dual interaction paradigm supports intuitive navigation of the formulation decision space.

**Figure 5:**
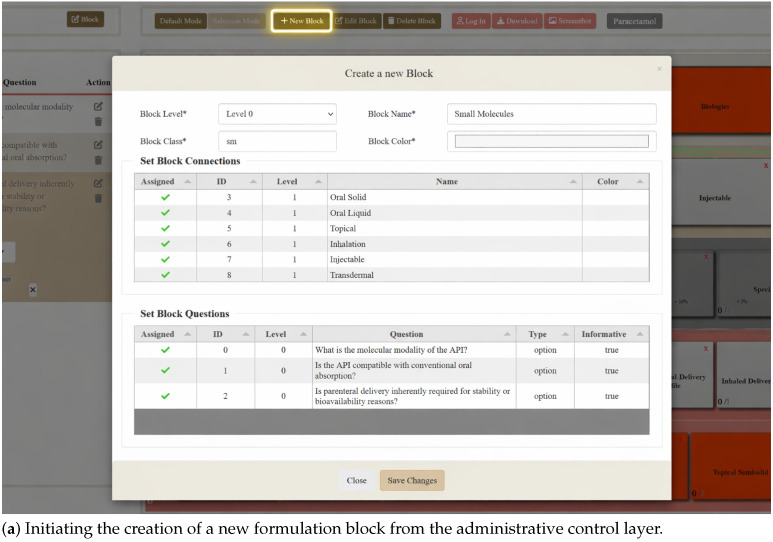

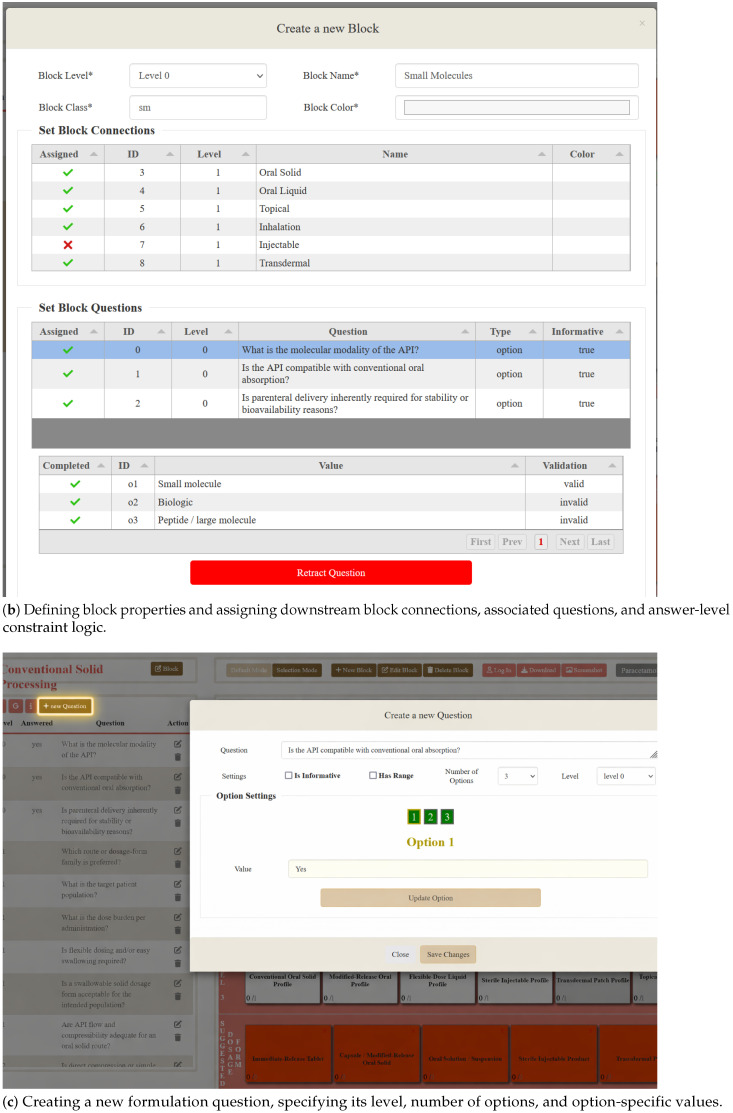
Administrative editing workflow of the Formulation tool. (**a**) A new block can be initiated from the administrative control layer. (**b**) The block-creation dialog allows the definition of block metadata and assignment of connected downstream blocks, associated questions, and corresponding constraint logic. (**c**) New questions can be created and configured by specifying their level, settings, and option values. Together, these functions enable controlled extension and refinement of the hierarchical decision framework.

In addition to this core functionality, the tool provides several advanced features:

- Visualization of downstream formulation pathways originating from a selected block, enabling users to anticipate potential future decision options.
- Evaluation of complete formulation pathways using a multi-selection mechanism, allowing users to assess the feasibility of combinations of blocks across multiple decision levels.
- Context-aware display of all required questions associated with a selected set of blocks, supporting early identification of missing information or experimentally inaccessible parameters.
- An administrative interface that enables authorized users to create, modify, or remove formulation blocks, define connections between blocks, assign associated questions, and configure question options and validation logic. This functionality supports the controlled evolution of the underlying decision framework based on expert knowledge and new development findings (Figure 5).
- Manual adjustment of block validation states using color-based annotations, enabling users to incorporate external evidence or expert judgment without directly answering all associated questions.
- Support for saving formulation projects under development, downloading them for local storage, and later re-uploading them to continue the decision process. This also enables project sharing among colleagues when collaborative review or continued development is required.
- Support for attaching supplementary documents to individual formulation blocks, including images, experimental records, PDF files, and presentation files, in order to justify and document formulation decisions at specific stages of the pathway.

In summary, the tool enables structured and interactive exploration of formulation strategies through explicit knowledge representation, while final formulation decisions remain under expert control. Machine learning-based recommendation functionalities are not part of the current version and are planned for future development.

#### 3.3.2. Procedure, Input, and Expected Results

The Formulation tool supports decision-making in complex, hierarchical formulation problems by guiding users through a sequence of formulation-relevant decision levels. In the context of the present work, the objective is to identify one or more feasible dosage form strategies for a given active pharmaceutical ingredient (API). To achieve this, the formulation process is explored from the initial level to the final decision level, with the goal of identifying at least one valid or conditionally feasible formulation pathway.

The primary input to the Formulation tool is a structured JSON file that encodes the formulation strategy. This file contains the definition of formulation blocks, associated questions (variables), admissible answers, and the relationships between these elements, including constraint definitions. The JSON-based representation enables a clear separation between the formulation knowledge base and the application logic, facilitating maintainability and extensibility.

During tool usage, users provide input by answering questions associated with formulation blocks or by manually adjusting block validation states based on external evidence or expert judgment. As inputs are provided, block feasibility is evaluated dynamically according to the underlying constraint satisfaction logic. Changes in feasibility status are reflected through visual indicators, enabling users to track progress toward identifying feasible formulation pathways.

The expected outcome of the procedure is the identification of one or more feasible formulation paths connecting the initial decision level to the final dosage form level. These paths serve as decision-support outputs that assist formulation scientists in selecting candidate formulation strategies for further experimental evaluation.

#### 3.3.3. User Interface and Usage of the Tool

An overview of the Formulation tool user interface is shown in Figure 2, illustrating the interactive components that support navigation of the formulation decision process. The interface is organized into two primary components: a graph-based representation of the formulation strategy and a table of associated questions (variables). The graph view, displayed on the right-hand side, represents formulation blocks arranged across horizontal levels that reflect the ordinal progression of the formulation process, from early-stage API considerations to final dosage form selection. This hierarchical organization follows a typical multi-stage formulation development workflow used in pharmaceutical research.

The question table, shown on the left-hand side of the interface, lists the variables associated with the formulation blocks. These variables represent formulation-relevant parameters and decision criteria that must be evaluated during the formulation process. Selecting a block in the graph dynamically highlights the relevant questions in the table, while selecting a question highlights all associated blocks. This bidirectional interaction supports intuitive exploration of the relationships between formulation options and required input parameters.

Users interact with the tool by providing responses to questions or by manually assigning validation states to formulation blocks based on external evidence or expert knowledge. As inputs are provided, block validity is updated in real time according to the underlying constraint satisfaction logic. Valid, conditionally feasible, and invalid blocks are visually distinguished using color-coded indicators, allowing users to quickly assess the feasibility of formulation pathways.

The interface supports both forward exploration and backward revision of decisions. Users may revisit earlier levels, modify inputs, and explore alternative formulation pathways without losing previously evaluated information. This interaction paradigm enables iterative refinement of formulation strategies and supports transparent, traceable decision-making throughout the formulation process.

In practice, the tool is used by formulation experts to explore feasible formulation strategies, identify critical information gaps, and compare alternative pathways before committing to experimental studies. Users may also save and reload ongoing projects, share them with collaborators, and attach supporting documents to selected formulation blocks in order to preserve justification and supporting evidence throughout the development process.

#### 3.3.4. Interactive Visualization

The Formulation tool is designed as an interactive visualization system to support active user engagement throughout the formulation decision process. All major elements of the formulation strategy, including formulation blocks, hierarchical levels, and associated questions, are directly interactive within the user interface.

Interaction with the tool enables users to explore relationships between formulation options and required input parameters. Selecting a formulation block highlights its associated questions, while selecting a question highlights all formulation blocks influenced by that variable. This bidirectional interaction facilitates understanding of dependencies and constraints within the formulation strategy.

As users provide responses to questions, the validation status of affected blocks is updated in real time and communicated through color-coded visual indicators. These indicators distinguish valid, conditionally feasible, and invalid blocks, allowing users to quickly assess the impact of their inputs on the overall feasibility of formulation pathways.

The interface supports iterative exploration by allowing users to revisit earlier decisions, modify inputs, and explore alternative formulation paths without restarting the process. The structured yet interactive design enables formulation scientists to efficiently observe, track, and manage the formulation decision process without requiring specialized training beyond domain expertise in pharmaceutical formulation.

### 3.4. Expert Evaluation of the Tool

To assess the practical applicability of the proposed decision-support framework, a structured evaluation was conducted using interaction logs from five expert users. Each user completed six predefined formulation scenarios, resulting in a total of 30 task runs.

The scenarios were based on three representative drugs—amoxicillin, paracetamol, and ibuprofen—each evaluated under adult and pediatric use conditions:

(i) Amoxicillin, adult use;
(ii) Amoxicillin, pediatric use;
(iii) Paracetamol, adult use;
(iv) Paracetamol, pediatric use;
(v) Ibuprofen, adult use;
(vi) Ibuprofen, pediatric use.

The scenarios were selected to assess whether the tool reproduces formulation-family choices consistent with established pharmaceutical practice, particularly the distinction between oral solid adult formulations and oral liquid pediatric formulations. In addition, the scenarios enabled analysis of how users navigate the constraint-based decision structure and how formulation pathways are explored across different levels of the decision hierarchy.

Users were instructed to interact with the tool until at least one feasible formulation pathway had been identified. Complete traversal of all branches was not required, reflecting realistic expert usage in which decision-support tools are used to identify plausible solutions efficiently.

All interactions were recorded in structured JSON format, capturing timestamps, selected answers, affected formulation blocks, and resulting validity states. These logs enabled reconstruction of decision pathways and quantitative analysis of interaction behavior.

Quantitative endpoints included: (i) number of answered questions, (ii) number of unique blocks traversed, (iii) completion time, (iv) number of terminal outputs reached, and (v) agreement with expected formulation-family outcomes. The analysis was descriptive, focusing on means, medians, ranges, and scenario-level comparisons.

## 4. Case Study

While the formulation strategy implemented in the current version of the tool is primarily derived from oral solid dosage form development practices, the underlying framework is general and can be extended to other formulation types. The case study therefore includes examples beyond oral solid dosage forms to illustrate the flexibility and adaptability of the proposed decision-support approach across different formulation contexts. This section presents a case study in pharmaceutical drug formulation to illustrate the application and capabilities of the proposed decision-support tool. The case study is intended to demonstrate how the tool implements formulation strategy knowledge and supports expert decision-making in a complex, hierarchical problem setting.

Drug formulation is a multi-stage process that requires the consideration of numerous interdependent factors, including physicochemical properties of the active pharmaceutical ingredient (API), biopharmaceutical requirements, manufacturability, regulatory constraints, and patient-related considerations. Decisions made at early stages of development can substantially influence downstream formulation options, processing routes, and final product characteristics.

The proposed tool supports this end-to-end formulation process by guiding users through a structured sequence of decision levels. Starting from API-related considerations, the tool assists users in exploring feasible formulation strategies through successive stages, including route and dosage-form family selection, manufacturing strategy selection, and product profile definition, ultimately converging to a suggested dosage form. At each stage, formulation options are evaluated against explicitly encoded constraints and expert-defined criteria, allowing users to systematically narrow the decision space while retaining flexibility to explore alternative pathways.

The underlying framework represents formulation development as a constraint-driven, hierarchical decision process in which each decision level refines the feasible solution space. Rather than enforcing a single deterministic pathway, the system preserves multiple valid and conditionally feasible alternatives, enabling users to explore formulation strategies in a manner consistent with real-world expert reasoning.

The case study applies the tool to representative drug formulation scenarios to assess its suitability for supporting formulation decision-making across these interconnected stages. The focus is placed on how the tool facilitates a structured exploration of formulation pathways, highlights feasibility constraints at different decision levels, and improves transparency and traceability throughout the formulation process. The following subsections describe the formulation context, the integration of the case study into the tool, the experimental setup, and observations derived from expert interaction with the system.

### 4.1. Background on Drug Formulation

Drug formulation is the process of designing a pharmaceutical product that delivers an active pharmaceutical ingredient (API) to the patient in a suitable dosage form while ensuring safety, efficacy, and quality. Formulation scientists must balance multiple considerations, including bioavailability, stability, release characteristics, patient population, manufacturability, and regulatory requirements, to achieve the intended therapeutic outcome.

The choice of formulation strategy depends strongly on the physicochemical and biopharmaceutical properties of the API, as well as the intended route of administration and clinical use. Conventional oral solid dosage forms, such as tablets and capsules, remain the most widely used and typically involve formulation decisions related to excipient selection, compression behavior, and manufacturability. Liquid formulations, including solutions and suspensions, are often preferred for pediatric or other populations requiring flexible dosing and easier administration, but they introduce additional challenges related to stability, dose uniformity, and product handling.

Across formulation types, development decisions are hierarchical and interdependent. Early choices, such as target population or route-related suitability, constrain later formulation possibilities and influence downstream decisions. As a result, formulation development represents a complex decision-making problem in which structured evaluation of alternatives may improve transparency, consistency, and efficiency.

In the present case study, drug formulation is used as a representative application domain to demonstrate how the proposed decision-support tool can assist formulation scientists in navigating these interconnected decisions. Rather than focusing on a proprietary development program, the case study uses representative drugs with well-established dosage-form families in public pharmaceutical practice, thereby enabling scenario-based evaluation of the tool against realistic formulation expectations.

### 4.2. Overview of the Process

The formulation process addressed in this case study follows a structured, multi-stage workflow that reflects common reasoning steps in pharmaceutical development. In the current implementation, the process is represented as a sequence of hierarchical decision levels, each corresponding to a specific formulation stage with associated constraints and decision criteria:

(i) **API modality selection (Level 0):** The decision process begins by characterizing the molecular modality of the Active Pharmaceutical Ingredient (API), distinguishing, for example, between small molecules and biologics. These properties impose fundamental constraints on feasible formulation routes and downstream processing strategies.
(ii) **Route and dosage-form family selection (Level 1):** Based on API characteristics and user-defined requirements such as target population, dose burden, and administration constraints, alternative route and dosage-form families are evaluated (e.g., oral solid, oral liquid, topical, inhalation, injectable, transdermal).
(iii) **Manufacturing strategy selection (Level 2):** For each feasible dosage-form family, appropriate manufacturing strategies are assessed, including conventional solid processing, wet or liquid processing, sterile processing, and specialized fabrication approaches. This level reflects technical feasibility and process-related constraints.
(iv) **Product profile selection (Level 3):** Candidate formulation profiles are refined further by evaluating product-specific requirements such as release characteristics, delivery mechanism, stability considerations, and therapeutic objectives (e.g., immediate-release oral solid, modified-release formulations, injectable profiles, or transdermal systems).
(v) **Final dosage-form output (Level 4):** The final level represents concrete dosage-form recommendations derived from the selected formulation pathway, such as tablets, capsules, oral liquids, injectables, transdermal patches, or inhaled products.
(vi) **Constraint-driven feasibility evaluation:** At each level, candidate pathways are dynamically evaluated as valid, invalid, or conditionally feasible based on the degree to which the encoded formulation constraints are satisfied. These evaluations propagate through the decision graph, guiding users toward feasible formulation pathways.

This hierarchical structure reflects the interdependencies between formulation decisions and provides the basis for representing the formulation problem as a constraint-based decision process rather than as a single isolated classification task. This level-based structure directly corresponds to the hierarchical decision graph implemented in the tool (Figure 4).

### 4.3. Case Study Integration into the Tool

The formulation process described above was formalized as a constraint satisfaction problem and implemented within the Formulation tool. The system is structured as a hierarchical, multi-level decision framework in which each formulation stage is represented as a decision level consisting of interconnected formulation blocks. These blocks encode formulation options and are evaluated based on explicitly defined constraints derived from formulation knowledge and expert input. Decisions propagate across levels, progressively refining the feasible solution space from high-level formulation choices to final dosage-form suggestions.

Each block can exist in one of three validation states—valid, invalid, or conditionally feasible—as defined in the constraint-evaluation framework described above. In the underlying JSON representation of the tool, the intermediate conditionally feasible state is labeled maybe_valid.

Users interact with the tool by answering formulation-relevant questions at each decision level. These interactions dynamically update block validity and determine which formulation pathways remain feasible. For example, selecting a pediatric target population constrains the available dosage form options, guiding users toward oral liquid formulations rather than injectable routes, while adult-targeted formulations permit a broader range of administration methods.

In addition to question-based constraint evaluation, the tool provides an explicit block-validation mechanism that allows users to manually confirm the validity of a formulation block. This functionality enables expert users to proceed to subsequent decision levels without answering all associated questions when sufficient domain knowledge is available. As observed in the evaluation, this feature supports expert-driven decision-making but may also lead to partially evaluated constraint states when intermediate validity is interpreted as sufficient for progression.

This interaction paradigm illustrates how the tool supports expert reasoning by making dependencies between formulation decisions explicit. Rather than replacing expert judgment, the system facilitates structured navigation of formulation options and helps prevent logically inconsistent decision combinations across different stages of development, while also accommodating non-linear and exploratory interaction patterns.

The constraint logic implemented in the tool is initially defined by an expert team involved in pharmaceutical formulation and drug development. These experts formalize domain knowledge into structured decision rules, including formulation blocks, inter-level relationships, and constraint definitions associated with each question and answer. This ensures that the decision framework reflects established formulation principles and industrial best practices.

The system is designed to support iterative refinement of this knowledge base. An administrative expert role is responsible for updating and extending the decision logic based on new experimental findings, formulation strategies, or regulatory considerations. Administrative users can modify the structure of the decision framework by adding or removing levels and formulation blocks, defining or updating relationships between blocks, and creating new formulation-relevant questions. In addition, they can assign validity states to possible answers, thereby encoding new constraint logic into the system.

In contrast, standard expert users interact with the tool only at the decision-support level and do not have access to modify the underlying logic. This separation ensures consistency of the decision framework while allowing controlled evolution of formulation knowledge over time.

### 4.4. Task Scenarios

To evaluate and validate the tool under realistic but controlled conditions, six predefined formulation scenarios were constructed using three representative drugs under adult and pediatric use conditions: amoxicillin, paracetamol, and ibuprofen. These drugs were selected because their dosage-form families are widely established in public pharmaceutical practice and therefore provide a suitable basis for assessing whether the tool identifies pharmaceutically plausible formulation pathways.

The six scenarios were:

(i) Amoxicillin, adult use;
(ii) Amoxicillin, pediatric use;
(iii) Paracetamol, adult use;
(iv) Paracetamol, pediatric use;
(v) Ibuprofen, adult use;
(vi) Ibuprofen, pediatric use.

Before the evaluation, each user was individually introduced to the Formulation tool and its interaction workflow. The interface, block-selection mechanism, question-answering process, and progression across the hierarchical decision levels were demonstrated manually. Users were instructed that the goal of each task was to identify at least one feasible formulation path from Level 0 to the final dosage-form output. A task was considered complete once one feasible path had been found. Users were allowed, but not required, to continue exploring additional feasible pathways after this point.

The evaluation therefore distinguished between *user success* and *tool success*. User success was defined as the identification of at least one feasible path through the decision framework. Tool success was defined as the ability of the encoded constraint logic to guide users toward pharmaceutically plausible formulation pathways while blocking or penalizing incompatible combinations of formulation decisions.

Because formulation development may legitimately admit more than one feasible solution, the scenarios were not associated with a single predetermined correct pathway. Instead, each scenario was associated with an expected plausible formulation region. For adult scenarios, this plausible region was expected to favor oral solid formulation pathways; for pediatric scenarios, it was expected to favor oral liquid pathways, reflecting the importance of dosing flexibility and ease of administration in pediatric use. Downstream manufacturing strategies, product profiles, and final dosage-form outputs could vary, provided that the resulting path remained feasible and pharmaceutically plausible.

This study design allowed assessment not only of whether users could identify feasible formulation pathways, but also of how they interacted with the decision-support system, including navigation strategy, depth of constraint evaluation, and exploratory behavior across the hierarchical decision structure. Expected plausible formulation regions were defined using a combination of official marketed-product information and API-related formulation rationale from regulatory and scientific sources. The drugs amoxicillin, ibuprofen, and paracetamol/acetaminophen were treated as small-molecule APIs, consistent with publicly available chemical and pharmacological records [38,39,40].

Marketed-product information was then used to justify the dominant adult and pediatric formulation regions. Official labels document oral solid dosage forms for all three APIs, including amoxicillin tablets/capsules, ibuprofen tablets, and acetaminophen tablets [41,42,43]. The same sources also document oral liquid products such as amoxicillin oral suspension, ibuprofen oral suspension, and acetaminophen oral suspension [41,44,45]. The dominant adult formulation region was therefore defined as oral solid, whereas the dominant pediatric formulation region was defined as oral liquid.

This adult–pediatric distinction was further supported by regulatory and formulation guidance indicating that liquid dosage forms are particularly valuable in younger pediatric populations because they improve swallowability and allow greater dosing flexibility [46,47]. At a deeper formulation level, the selected plausible paths were additionally supported by API-specific rationale. Amoxicillin has established oral use and is stable in gastric acid with rapid oral absorption [48]. Ibuprofen is a small-molecule drug whose formulation strategy is strongly influenced by low aqueous solubility and biopharmaceutics classification system (BCS) class II behavior [49]. Acetaminophen/paracetamol has well-established immediate-release oral solid formulations while also remaining highly suitable for pediatric oral liquid use when flexible dosing is required [45,46,50]. On this basis, the expected outcomes used in the evaluation were interpreted not as single fixed correct pathways, but as dominant plausible formulation regions defined by marketed dosage-form reality, target-population suitability, and API-related formulation characteristics.

### 4.5. Evaluation Protocol and Success Criteria

The evaluation protocol was designed as a guided but non-exhaustive task-based study. Users were asked to identify at least one feasible formulation path from the initial API-modality level to the final dosage-form output. They were not required to exhaustively evaluate all remaining branches after one feasible path had been identified. This protocol reflects the realistic use of a formulation decision-support tool, where the immediate goal is often to identify one or more viable candidates for further experimental consideration rather than to enumerate all theoretically feasible solutions.

A task was considered *complete* when the user reached at least one terminal dosage-form output through a path that remained valid or conditionally feasible within the encoded decision framework. A path was considered *fully evaluated* when the relevant downstream blocks along the selected route had their associated questions resolved to a final validity state. In contrast, *partially evaluated feasible paths* were those in which users continued after observing intermediate valid or conditionally feasible states without completing all associated block-level questions.

For the purposes of the study, *user success* was defined as identifying at least one feasible formulation path. *Tool success* was defined as the ability of the system to constrain users to pharmaceutically plausible solution spaces by supporting feasible combinations of decisions while disfavoring or blocking incompatible combinations. Accordingly, the evaluation focused not only on terminal outputs, but also on whether the level-wise pathway selected by the user remained plausible with respect to molecular modality, route and dosage-form family, processing strategy, product profile, and final dosage-form output.

## 5. Evaluation Results

### 5.1. Quantitative Results

To interpret user performance beyond simple route-level agreement, each scenario was associated with an expected plausible formulation region defined across the implemented decision levels of the tool. These regions did not represent a single predetermined correct pathway, but rather a set of pharmaceutically plausible level-wise outcomes consistent with the intended population, dose burden, and downstream formulation logic encoded in the system. Table 2 and Table 3 summarize these expected plausible regions for the six study scenarios across the implemented decision levels of the tool.

Across the 30 evaluated sessions (five expert users, six scenarios each), all runs reached at least one terminal formulation output, corresponding to a completion rate of 100%. On average, each session included 12.3 answered questions (median: 13; range: 4–17), traversed 10.5 unique formulation blocks, and lasted 129.5 s (median: 113.5 s). The mean number of terminal outputs per session was 1.43, indicating that most runs converged to a primary solution, while a subset of sessions explored additional feasible pathways.

At the scenario level, the tool consistently identified formulation pathways aligned with expected dosage-form families. Adult scenarios predominantly converged to oral solid pathways, whereas pediatric scenarios favored oral liquid pathways, reflecting established pharmaceutical practice.

However, differences in user interaction behavior influenced the degree of pathway completeness and exploration. While some users followed a structured, top-down approach and evaluated most relevant constraints, others adopted a more exploratory interaction style and terminated the process after identifying a valid formulation route. As a result, evaluation outcomes are reported in terms of both route-level agreement and pathway completeness. A quantitative summary of these interaction metrics across the six formulation scenarios is provided in Table 4.

Observed user outcomes were further examined at the level of downstream formulation regions and terminal dosage-form outputs. Because multiple feasible paths could reasonably exist within the expected plausible region of a scenario, the results were interpreted in terms of dominant observed regions, alternative plausible branches, and pathway completeness. Table 5 summarizes these dominant observed outcomes across the six formulation scenarios.

Across all scenarios, route-level agreement with expected formulation outcomes was consistently achieved. However, downstream analysis showed that user convergence should not be interpreted as matching a single fixed path. Instead, users generally remained within the expected plausible formulation region, while differences between scenarios were primarily observed in the extent of alternative-path exploration and in whether the identified feasible path was fully or only partially evaluated.

### 5.2. Pathway Completeness and Exploration Behavior

Although all sessions reached at least one feasible terminal output, full evaluation of all downstream constraints was not uniformly observed. The study protocol defined task completion as identification of at least one feasible path from Level 0 to the final dosage-form output. Consequently, users were not required to exhaustively evaluate all remaining branches once a feasible route had been identified.

To distinguish between different completion patterns, runs were interpreted according to three pathway-status categories: (i) *complete paths*, in which the selected downstream blocks were evaluated to a final validity state; (ii) *partially evaluated feasible paths*, in which users reached a feasible terminal output but did not complete all associated downstream block-level questions; and (iii) *exploratory multi-output runs*, in which users continued beyond the first feasible path and inspected additional feasible terminal outputs. Table 6 summarizes these pathway-completion patterns at the scenario level.

Approximately one-third of sessions produced more than one terminal output, indicating deliberate exploration of alternative feasible branches. This behavior was most evident in adult amoxicillin and pediatric ibuprofen scenarios, where users frequently explored additional outputs beyond the first identified path.

Importantly, incomplete downstream evaluation did not imply incorrect pathway selection. In most cases, users terminated interaction after identifying a feasible formulation route within the expected plausible region. This distinction highlights the difference between *functional completion*, defined by the evaluation protocol as identification of one feasible path, and *full pathway validation*, which would require complete downstream resolution of all selected blocks.

### 5.3. Levels and Block Traversal

Analysis of the interaction logs showed consistent traversal of the upper levels of the decision hierarchy across all scenarios. All sessions passed through Level 0 (API modality selection) and Level 1 (route and dosage-form family selection), confirming that the high-level constraint logic of the tool was systematically engaged. In the present case-study scenarios, the dominant Level 0 branch was *Small Molecule*, followed at Level 1 by either *Oral Solid* or *Oral Liquid*, depending on the intended adult or pediatric formulation context.

Differences became more apparent at the downstream levels, particularly at Level 2 (manufacturing strategy selection) and Level 3 (product profile selection). Adult scenarios more frequently activated blocks associated with *Conventional Solid Processing* and downstream oral solid profiles, whereas pediatric scenarios more frequently activated *Wet/Liquid Processing* and liquid-compatible downstream profiles. This pattern is consistent with the expected plausible formulation regions defined for adult and pediatric use cases.

The interaction logs further showed that users did not always converge to the same downstream profile, even when they remained within the same plausible solution region. In some sessions, users followed the dominant expected path to a conventional downstream profile, whereas in others, they traversed alternative but still feasible profile blocks that preserved a plausible terminal dosage-form output. These observations indicate that the system constrains users to pharmaceutically reasonable regions of the decision graph while still allowing non-identical downstream trajectories.

Occasional traversal of additional blocks reflected exploratory inspection of alternative feasible pathways rather than inconsistency in the implemented logic. The observed behavior therefore supports the interpretation that the hierarchical structure of the tool effectively guides users from general formulation constraints to progressively more specific downstream decisions, while still preserving feasible alternative branches when appropriate.

**Figure 6:**
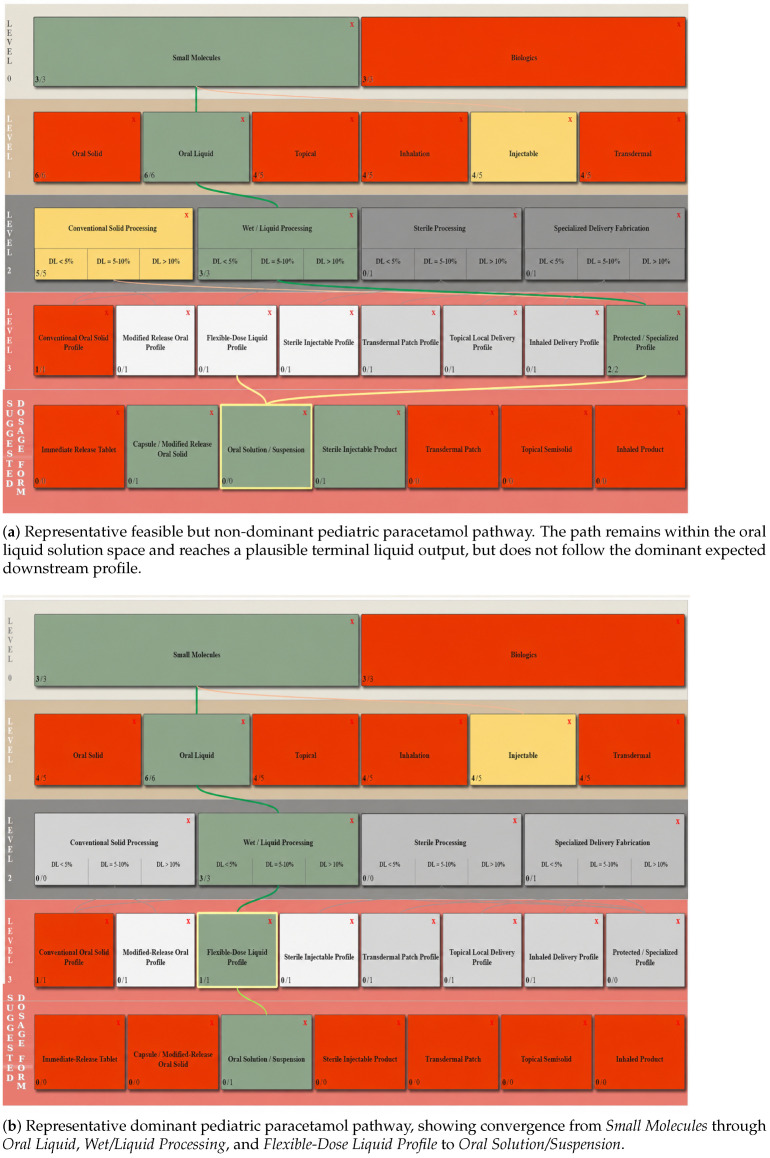
Representative examples of pediatric paracetamol evaluation sessions. Both examples converge within the expected oral liquid feasible region, but differ in downstream profile selection. Panel (**a**) illustrates an alternative but still plausible path, whereas panel (**b**) shows the dominant expected pathway. Overall, the examples demonstrate how the tool preserves multiple feasible solution branches while still guiding users toward pharmaceutically plausible outputs.

Representative examples of level-wise pathway propagation are shown in Figure 6. Panel (A) illustrates a feasible but non-dominant pediatric paracetamol pathway, whereas panel (B) shows the dominant expected pediatric liquid pathway converging from *Small Molecules* through *Oral Liquid*, *Wet/Liquid Processing*, and *Flexible-Dose Liquid Profile* to *Oral Solution/Suspension*.

### 5.4. Qualitative Observations

A key qualitative finding of the evaluation was the consistent separation between adult and pediatric formulation logic. Across all drugs, adult scenarios converged toward oral solid formulation regions, whereas pediatric scenarios converged toward oral liquid formulation regions. This indicates that the encoded constraints related to swallowability, dosing flexibility, and administration suitability operate as intended within the evaluated scenarios.

The evaluation also demonstrated that the tool preserves uncertainty where appropriate. In several scenarios, users encountered both valid and conditionally feasible pathways, allowing exploration of alternative formulation strategies. This behavior reflects the intended expert-in-the-loop design of the system and distinguishes it from deterministic decision-support approaches.

The pediatric paracetamol sessions further illustrated that users could reach either the dominant expected flexible-dose liquid pathway or an alternative but still plausible liquid-oriented pathway. This indicates that the tool preserved feasible solution diversity while constraining users to pharmaceutically reasonable regions rather than forcing single downstream solutions.

In a subset of runs, users reached the expected plausible formulation region but did not fully evaluate downstream product-profile blocks. This suggests that users considered the task sufficiently resolved once a plausible formulation route had been identified. Such behavior is consistent with real-world formulation practice, where decision-making is often iterative and does not necessarily involve an exhaustive evaluation of all constraints.

Closer inspection of the interaction logs indicates that, in some cases, users discontinued answering additional questions within a selected block after observing an initial valid or conditionally feasible state triggered by a single response. As a result, the full set of constraints associated with that block was not evaluated, and downstream feasibility was determined based on partially resolved information.

Although the tool provides an explicit block-validation mechanism that allows expert users to manually confirm block feasibility and intentionally bypass remaining questions, this functionality was not part of the instructed evaluation protocol. The observed behavior therefore reflects the interpretation of intermediate validity states as sufficient decision signals, rather than a deliberate use of the expert-override feature.

### 5.5. User Interaction Patterns

User interaction logs revealed two dominant navigation styles across the evaluation sessions: a *structured* mode of interaction and a more *exploratory* mode of interaction.

In structured sessions, users progressed sequentially from the upper levels of the hierarchy toward the final dosage-form output. These runs more often began at Level 0, followed the intended top-down logic of the decision graph, and resulted in more complete evaluation of downstream constraints. Structured sessions therefore tended to produce more fully resolved formulation pathways.

In exploratory sessions, users interacted with the graph in a less linear manner. Some users began at intermediate levels rather than at the initial API-modality layer, selected familiar downstream formulation blocks first, or inspected alternative branches before resolving all upstream and downstream constraints. In these runs, users often terminated the task once a feasible route had been identified, resulting in partially evaluated but still plausible formulation paths.

Despite these differences in navigation strategy, both interaction modes generally converged within the expected plausible formulation regions of the scenarios. This indicates that the tool supports flexible usage styles while preserving logical consistency of the underlying formulation framework.

These observations suggest that the Formulation tool accommodates both systematic and non-linear reasoning strategies. This flexibility is important for practical adoption, as formulation experts may approach decision problems differently depending on prior experience, familiarity with the API, and the extent to which they wish to explore alternative feasible paths before selecting a candidate formulation for experimental follow-up.

### 5.6. Exploratory Behavior and Pathway Completeness

In addition to broad interaction styles, the evaluation logs revealed measurable differences in the extent to which users explored the decision space and fully resolved the selected formulation pathway.

Across multiple sessions, users demonstrated iterative interaction behavior, including revisiting previously answered questions, changing responses, and inspecting alternative downstream branches before stopping. These observations indicate that the tool was used not only as a linear decision aid, but also as an exploratory reasoning environment in which users could compare plausible formulation options before deciding whether further evaluation was necessary.

Three pathway-completion patterns were observed:

- **Complete paths:** Users evaluated the selected route through the downstream blocks to a final validity state, resulting in a fully resolved formulation pathway.
- **Partially evaluated feasible paths:** Users reached a feasible terminal output but did not answer all associated questions for the selected downstream blocks, leaving some parts of the path only partially resolved.
- **Exploratory multi-output runs:** Users continued beyond the first feasible path and inspected one or more additional feasible terminal outputs within the same plausible formulation region.

The second pattern reflected early stopping after identification of a plausible route rather than explicit use of the manual block-validation function. Although the tool provides a block-validation mechanism that can be used by experienced users to confirm feasibility and bypass remaining questions, this feature was not part of the instructed evaluation protocol and was not required for task completion in the present study.

From an analytical perspective, partially evaluated feasible paths should therefore not be interpreted as incorrect outcomes. Instead, they reflect the study protocol and a realistic usage pattern in which users stop once one viable formulation candidate has been identified. At the same time, these runs provide less complete downstream information than fully evaluated paths and therefore reduce the resolution at which final pathway agreement can be assessed.

Table 7 summarizes the qualitative relationship between answer revision behavior, degree of downstream constraint completion, and the resulting interpretation of each interaction pattern.

Overall, these findings demonstrate that the tool supports both an efficient identification of feasible formulation candidates and a broader exploration of alternative plausible branches. This dual behavior is consistent with real-world formulation practice, in which experts may either stop after finding one viable route or continue exploring additional options before selecting a candidate pathway for experimental follow-up.

## 6. Discussion

This section discusses the observations derived from the case study and reflects on the methodological contributions of the proposed decision-support approach. The focus is placed on the novelty of formalizing formulation strategy knowledge into an interactive, constraint-based system and on how this approach differs from conventional formulation practices.

### 6.1. Innovation in Drug Development

The primary innovation of this work lies in the formalization of formulation strategy knowledge into a structured decision-support framework that makes formulation reasoning explicit, transparent, and computationally navigable. Rather than treating formulation development as an ad hoc sequence of disconnected expert decisions, the proposed approach organizes the process into a hierarchical constraint-based system in which upstream choices progressively restrict or preserve downstream formulation possibilities.

This work builds on established formulation-strategy concepts by demonstrating how expert knowledge can be translated into an interactive digital framework that supports systematic exploration of feasible formulation pathways. By explicitly representing decision stages, interdependencies between formulation choices, and block-level feasibility states, the approach provides a structured alternative to experience-driven formulation workflows commonly used in early development.

An important aspect of pharmaceutical formulation development is the need to integrate patient-related and product-related constraints into the decision process. Differences in target population, dose burden, swallowability requirements, dosing flexibility, stability, and route suitability can substantially influence the set of acceptable formulation options. In the present work, these considerations are represented through explicitly encoded constraint logic, allowing such factors to shape the feasible formulation region without forcing a single predetermined outcome.

Dose-related and patient-related considerations play an important role in pharmaceutical formulation design and are partially reflected in the current framework through expert-defined qualitative constraints, such as target population, swallowability, administration suitability, and dosing flexibility. However, the present implementation does not perform quantitative dose optimization or individualized patient-response modeling. These aspects would require additional pharmacokinetic, pharmacodynamic, and patient-specific modeling layers and therefore represent an important direction for future development.

The evaluation supports this interpretation. Across all six predefined scenarios, users consistently converged within the expected adult oral solid or pediatric oral liquid formulation regions, demonstrating that the implemented logic captured clinically and pharmaceutically meaningful distinctions at the route-family level. At the same time, the results showed that multiple downstream pathways could remain feasible within the same plausible solution region. This indicates that the framework does not merely reproduce one fixed recommendation, but rather preserves the diversity of viable formulation options that experts may wish to compare before selecting a candidate for experimental follow-up.

A further important innovation is the distinction between user success and tool success. In the present study, user success was defined as identifying at least one feasible path through the decision framework, whereas tool success was defined by the ability of the encoded constraint logic to guide users toward pharmaceutically plausible pathways while disfavoring incompatible combinations of decisions. This dual evaluation perspective is especially relevant in formulation development, where multiple acceptable solutions may exist and where decision-support systems should help define a plausible solution space rather than enforce a single deterministic answer.

Finally, the observed variability in interaction behavior further supports the interpretation of the tool as an exploratory expert-in-the-loop system. Some users followed a structured top-down workflow, whereas others navigated the graph more selectively and stopped after identifying one viable route. The fact that both styles generally remained within plausible formulation regions suggests that the framework is sufficiently structured to preserve logical consistency while remaining flexible enough to accommodate realistic expert reasoning.

### 6.2. Digital Tool Development

A key outcome of this research is the development of the Formulation tool, a digital, interactive visualization system that implements the formulation strategy within a constraint-based decision framework. The tool translates formulation knowledge into an explicit and navigable structure, enabling users to explore formulation options and assess feasibility in a transparent manner.

By providing a graphical representation of formulation pathways and their associated constraints, the tool enhances accessibility and usability of the formulation strategy. The digital format supports consistent application of formulation knowledge across projects and facilitates documentation and communication of formulation decisions among stakeholders.

### 6.3. Methodological Differences

Methodologically, this work differs from existing decision-support approaches in pharmaceutical formulation by combining a hierarchical decision graph with a constraint-based evaluation framework. While many existing systems focus either on static rule-based decision trees or on data-driven prediction models, the proposed approach emphasizes interactive exploration of feasible formulation regions under explicitly encoded constraints.

A central methodological feature of the framework is that it does not aim to identify a single optimal or universally correct formulation path. Instead, it is designed to represent a structured solution space in which multiple downstream pathways may remain feasible depending on user input, constraint resolution, and the degree of downstream evaluation. This is particularly important in formulation development, where more than one pharmaceutically acceptable solution may exist for the same API and target population.

The use of valid, invalid, and conditionally feasible states allows the system to represent both feasibility and uncertainty. Rather than collapsing uncertainty into a binary yes/no decision, the framework preserves intermediate states and therefore enables users to continue exploring plausible branches even when full downstream resolution has not yet been achieved. This behavior was reflected in the evaluation, where users could converge either to the dominant expected pathways or to alternative but still plausible formulation paths within the same solution region.

Another important methodological characteristic is the explicit distinction between pathway identification and pathway resolution. A user may identify a feasible path from the upper levels of the hierarchy to a terminal dosage-form output without necessarily resolving all associated downstream block-level questions. The framework therefore supports both *functional completion*, defined as the identification of at least one feasible formulation route, and *full pathway validation*, defined as complete downstream evaluation of the selected route. This distinction is especially relevant in realistic expert workflows, where the immediate goal is often to identify one viable candidate for experimental follow-up rather than to exhaustively resolve every remaining branch.

A further distinguishing feature of the proposed approach is its expert-in-the-loop character. The tool does not replace expert judgment, but augments it by structuring the formulation process and enforcing logical consistency across decision stages. Users remain free to explore alternative feasible branches, revise earlier selections, and stop once one acceptable route has been identified, while the system maintains explicit representation of dependencies between molecular modality, route family, processing strategy, product profile, and final dosage-form output.

Taken together, these methodological characteristics position the Formulation tool not as a deterministic recommendation engine, but as an interactive framework for constraint-guided exploration of pharmaceutically plausible solution spaces. This distinguishes it from systems that aim primarily at single-output prediction or automatic optimization.

### 6.4. Future Directions

The present work represents an initial step toward a formalized, interactive decision-support framework for pharmaceutical formulation. Future development of the tool will focus on extending its capabilities through the integration of machine learning techniques that learn from historical user interactions and support recommendation of formulation pathways. These extensions are intended to complement, rather than replace, expert decision-making.

In addition, the applicability of the approach will be explored in other formulation-intensive domains, such as cosmetics and soap manufacturing, to assess its generalizability beyond pharmaceutical drug development. Broader evaluation involving multiple users and formulation scenarios will be required to further assess usability, scalability, and impact in industrial settings.

### 6.5. Scalability and Adaptability

A key practical consideration for real-world deployment is the scalability of the tool as the number of formulation blocks, hierarchical levels, questions, and constraints increases. The current system is designed as a modular hierarchical decision framework in which organizations can extend or modify the formulation graph by adding new blocks, editing existing constraints through the administrative interface, or replacing the underlying JSON structure entirely with an alternative domain-specific decision model. In principle, this architecture is not restricted to a fixed number of levels or blocks and can accommodate substantially larger decision graphs than those evaluated in the present study.

From a structural perspective, increasing the number of blocks or constraints primarily affects the size of the search and validation space rather than the conceptual validity of the framework itself. Because constraint evaluation is performed locally on the basis of user selections and associated block logic, the system remains interpretable even as the graph expands. In practice, however, larger formulations involving, for example, hundreds of blocks and several hundred constraints may affect response time, interface usability, and maintenance effort, particularly if many interdependent rules must be evaluated simultaneously.

At the current stage, the tool has been validated functionally in the pharmaceutical formulation setting presented here, and the same underlying framework has also been adapted to other hierarchical decision scenarios, including cosmetics-related workflows such as soap formulation and non-pharmaceutical planning tasks such as travel design. These early adaptations support the generality of the framework and indicate that the approach is not limited to oral solid dosage-form development. Instead, the tool should be understood as a general constraint-based decision-support framework that can be configured for different application domains by redefining the block structure, decision levels, and associated constraint logic.

Although the present study focused on correctness, interpretability, and practical usability rather than on computational stress testing, the modular architecture is compatible with future large-scale expansion. For institutional deployment, each adopting organization can integrate its own in-house rules, proprietary formulation logic, and domain-specific constraints either directly through the administrative interface or by uploading a revised JSON configuration. This makes the framework scalable not only in size but also in scope, while preserving traceability and explicit control over the encoded decision logic.

## 7. Conclusions

This work presents a structured, constraint-based decision-support tool designed to assist formulation scientists in navigating the complexities of pharmaceutical formulation development. By encoding formulation knowledge into a hierarchical, interactive framework, the tool enables a transparent exploration of formulation pathways while ensuring that decisions remain traceable, reproducible, and grounded in explicitly defined constraints.

The evaluation suggests that the Formulation tool can guide users toward pharmaceutically plausible formulation regions across a range of representative adult and pediatric case-study scenarios. Adult tasks consistently converged within oral solid formulation regions, whereas pediatric tasks converged within oral liquid formulation regions, indicating that the encoded constraint logic captures clinically and pharmaceutically meaningful distinctions at the route-family level within this exploratory evaluation.

At the same time, the results showed that formulation success should not be interpreted as convergence to a single predetermined path. Multiple downstream pathways could remain feasible within the same plausible solution region, and users differed in the extent to which they fully resolved downstream constraints after identifying one viable route. This distinction motivated a separate interpretation of user success, defined as the identification of at least one feasible path, and tool success, defined as the ability of the decision framework to constrain users to pharmaceutically plausible pathways while disfavoring incompatible combinations of decisions.

The evaluation further showed that the tool supports both structured and exploratory modes of use. Some users followed the intended top-down progression through the hierarchy, whereas others entered the decision graph more selectively and stopped after one acceptable path had been identified. Despite these differences in navigation strategy, user trajectories generally remained within plausible formulation regions, indicating that the framework is sufficiently structured to preserve logical consistency while remaining flexible enough to support realistic expert reasoning.

The proposed framework suggests that formulation-strategy knowledge can be formalized as an interactive constraint-based system for pathway exploration rather than as a deterministic single-output recommendation. The architecture is designed to scale beyond the relatively small evaluation graphs used in the present study, as decision structures can be extended through additional blocks, levels, and constraints or replaced entirely through alternative JSON-based knowledge models. This provides a basis for future work, including broader evaluation, refinement of downstream constraint logic, integration of data-driven recommendation components that complement rather than replace expert decision-making, and systematic large-scale performance benchmarking.

## 8. Limitations and Future Directions

While this work demonstrates the feasibility and practical relevance of a constraint-based decision-support approach for pharmaceutical formulation development, several limitations should be acknowledged.

First, the evaluation was conducted on a limited number of scenarios and users and should therefore be interpreted as an exploratory study rather than as a definitive large-scale validation. Although the analysis included both quantitative interaction metrics and qualitative interpretation of pathway selection, broader studies involving additional experts, APIs, and formulation contexts will be required to assess generalizability, robustness, and statistical stability.

In addition, the present evaluation did not include a direct comparison against a manual non-tool formulation workflow, which would be valuable in future studies to assess the practical added value of tool-supported decision-making more explicitly.

Second, the current evaluation focused primarily on whether users could identify at least one feasible path within a plausible formulation region. As a result, some runs reached valid terminal outputs without full downstream resolution of all associated block-level constraints. This behavior is informative because it reflects realistic expert usage, but it also limits the extent to which fine-grained downstream pathway agreement can be interpreted. Future studies should therefore distinguish more explicitly between functional task completion, full downstream pathway validation, and comparison of alternative feasible branches.

Third, the knowledge base of the tool is explicitly encoded from expert-defined formulation logic. This design is a strength in terms of transparency and traceability, but it also means that the scope and quality of decision support depend on the completeness and accuracy of the underlying knowledge representation. Expanding and refining the encoded rules, downstream profile logic, and constraint definitions will be important for improving coverage of more diverse formulation situations and for sharpening separation between dominant and alternative feasible paths.

A further limitation of the present work is that formal computational benchmarking under larger graph configurations was not the primary focus of the evaluation. Accordingly, the current study does not yet report systematic stress tests for scenarios involving, for example, large numbers of blocks, interdependent constraints, or more deeply branched decision graphs. Future work should therefore include dedicated performance benchmarks assessing response time, rule-evaluation efficiency, interface usability, and maintenance complexity as the graph size increases. Such analyses would help characterize practical upper limits and guide optimization for larger institutional deployments. At the same time, the framework is structurally extensible: organizations can add or edit blocks and constraints through the administrative interface or replace the underlying JSON structure entirely with an alternative domain-specific knowledge model.

Fourth, the present framework is not intended to identify a single optimal formulation. Instead, it supports a structured exploration of a constrained solution space in which more than one plausible pathway may remain feasible. While this is appropriate for early-stage formulation reasoning, future development could incorporate ranking, prioritization, or data-driven recommendation layers to support comparison of feasible candidates without eliminating expert oversight.

Finally, although the current case study is situated in pharmaceutical formulation, the underlying framework is not restricted to one specific product class or therapeutic context. Future work should evaluate how well the approach transfers to additional formulation domains, broader pharmaceutical development settings, and other knowledge-intensive decision problems in which hierarchical reasoning under explicit constraints is required.

These limitations reflect the current stage of the work as a structured, expert-guided, and exploratory decision-support framework. They also define a clear path for future development toward broader validation, richer downstream pathway analysis, and integration of complementary data-driven methods.

## Figures and Tables

**Table 1:** Qualitative comparison of selected decision-support approaches relevant to pharmaceutical formulation and drug development.

Criterion	SeDeM	Wiklund	Proposed Tool
Primary purpose	Powder suitability for direct compression	Strategic decision-making across drug development	Interactive exploration of formulation pathways
Scope	Tablet preformulation	Drug-development lifecycle	Configurable formulation processes
Knowledge representation	Material parameters and indices	Mechanistic and simulation models	Rule-based constraints (JSON expert knowledge)
Decision approach	Parameter-based assessment	Simulation-based evaluation	Constraint satisfaction (feasibility-driven)
Optimization	Limited	Scenario evaluation	No optimization (feasibility focus)
Interactivity	Limited	Limited	High (interactive navigation)
Transparency	Medium	Medium	High (explicit constraints)
Output	Suitability indices	Simulated outcomes	Valid/invalid/conditionally feasible pathways
Role of expert	Interprets outputs	Interprets results	Central decision-maker

**Table 2:** Expected plausible formulation regions across the upper decision levels of the tool.

Scenario	Population	Expected L0	Expected L1	Expected L2
Amoxicillin adult	Adult	Small molecule	Oral solid dominant	Conventional solid processing dominant
				Wet/liquid processing may also remain plausible
Amoxicillin pediatric	Pediatric	Small molecule	Oral liquid dominant	Wet/liquid processing dominant
Ibuprofen adult	Adult	Small molecule	Oral solid dominant	Conventional solid processing dominant
Ibuprofen pediatric	Pediatric	Small molecule	Oral liquid dominant	Wet/liquid processing dominant
Paracetamol adult	Adult	Small molecule	Oral solid dominant	Conventional solid processing dominant
Paracetamol pediatric	Pediatric	Small molecule	Oral liquid dominant	Wet/liquid processing dominant

**Table 3:** Expected plausible formulation regions across downstream profile selection and final dosage-form outputs.

Scenario	Expected L3	Typical Acceptable Final Output (s)
Amoxicillin adult	Conventional oral solid profile and/or modified-release oral profile	Immediate-release tablet Capsule/modified-release oral solid
Amoxicillin pediatric	Flexible-dose liquid profile dominant	Oral solution/suspension
Ibuprofen adult	Conventional oral solid profile and/or modified-release oral profile	Immediate-release tablet Capsule/modified-release oral solid
Ibuprofen pediatric	Flexible-dose liquid profile dominant	Oral solution/suspension
Paracetamol adult	Conventional oral solid profile dominant Modified-release oral profile may remain plausible	Immediate-release tablet Capsule/modified-release oral solid
Paracetamol pediatric	Flexible-dose liquid profile dominant Alternative liquid-compatible profiles may remain plausible if still leading to a feasible oral liquid output	Oral solution/suspension

**Table 4:** Quantitative summary of interaction metrics across formulation scenarios. Route agreement reflects convergence toward the expected dosage-form family, while the number of terminal outputs indicates the degree of exploratory behavior.

Scenario	Expected Family	Route Agreement	Mean Answers	Median Answers	Mean Time (s)	Mean Terminal Outputs
Amoxicillin adult	Oral solid	High	13.0	14	94.8	2.0
Amoxicillin pediatric	Oral liquid	High	12.0	13	136.0	1.0
Ibuprofen adult	Oral solid	High	11.2	13	198.8	1.2
Ibuprofen pediatric	Oral liquid	High	12.6	13	107.6	1.4
Paracetamol adult	Oral solid	High	12.4	13	114.0	1.4
Paracetamol pediatric	Oral liquid	High	12.8	14	124.8	1.6

**Table 5:** Dominant observed formulation regions, most frequent terminal outputs, and pathway-completion patterns across the six evaluation scenarios. “Alternative plausible paths” indicates whether users also reached additional feasible branches within the expected plausible region. “Pathway status” summarizes whether runs were predominantly fully evaluated or included a mixture of complete and partially evaluated feasible paths. IR: immediate release; MR: modified release.

Scenario	Dominant Observed Region	Most Frequent Output(s)	Alternative Plausible Paths	Pathway Status
Amoxicillin adult	Oral solid	IR tablet; capsule/MR oral solid	Present	Mixed
Amoxicillin pediatric	Oral liquid	Oral solution/suspension	Limited	Mostly complete
Ibuprofen adult	Oral solid	IR tablet; capsule/MR oral solid	Present	Mixed
Ibuprofen pediatric	Oral liquid	Oral solution/suspension	Present	Mixed
Paracetamol adult	Oral solid	IR tablet; capsule/MR oral solid	Limited	Mostly complete
Paracetamol pediatric	Oral liquid	Oral solution/suspension	Present	Mixed

**Table 6:** Scenario-level summary of pathway-completion patterns. “Complete paths” indicates runs in which downstream blocks along the selected route were evaluated to a final validity state. “Partial feasible paths” indicates runs that reached a feasible output without full downstream constraint completion. “Multi-output runs” indicates runs in which users explored additional feasible terminal outputs beyond the first identified path.

Scenario	Complete Paths	Partial Feasible Paths	Multi-Output Runs	Interpretation
Amoxicillin adult	Medium	Medium	High	Strong exploration of alternative solid-oriented outputs
Amoxicillin pediatric	High	Low	Low	Rapid convergence within the expected liquid region
Ibuprofen adult	Medium	Medium	Low–Medium	Feasible adult solid paths with moderate downstream variation
Ibuprofen pediatric	Medium	Medium	Medium	Pediatric liquid paths with broader exploratory behavior
Paracetamol adult	High	Low–Medium	Low	Stable convergence within the adult oral solid region
Paracetamol pediatric	Medium	Medium	Medium	Mixture of dominant and alternative liquid-compatible paths

**Table 7:** Observed exploratory and completion patterns across evaluation sessions. Patterns are distinguished by degree of answer revision, downstream constraint completion, and resulting formulation-pathway resolution.

Interaction Pattern	Answer Changes	Constraint Completion	Interpretation
Complete path	Low–Moderate	High	Fully resolved feasible pathway
Partially evaluated feasible path	Low–Moderate	Low–Medium	Feasible output reached before full downstream evaluation
Exploratory multi-output run	Moderate–High	Medium–High	Additional feasible branches inspected beyond the first valid path

## Data Availability

The JSON-based knowledge representation underlying the evaluated tool configuration, together with the JSON files recording the user interaction experiments analyzed in this study, are provided as Supplementary Material. To support transparency and reproducibility, the public source code repository is available at https://github.com/reihanehmanteghi91/drug-formulation (accessed on 28 April 2026), and a live deployed version of the application is accessible at https://019db467-c018-09fc-19d0-e4edc7395e43.share.connect.posit.cloud/ (accessed on 25 April 2026).

## Supplementary Materials

The following supporting information can be downloaded at: https://www.mdpi.com/article/10.3390/pharmaceutics18060635/s1, supplementary materials containing the JSON-based knowledge representation underlying the evaluated tool configuration and the JSON files recording the user-interaction experiments analyzed in this study.

## Abbreviations

The following abbreviations are used in this manuscript:

API	Active Pharmaceutical Ingredient
CSP	Constraint Satisfaction Problem
ML	Machine Learning
AI	Artificial Intelligence
ANN	Artificial Neural Network
PAT	Process Analytical Technology
JSON	JavaScript Object Notation
HPLC	High-Performance Liquid Chromatography
DM	DailyMed
EMA	European Medicines Agency
BCS	Biopharmaceutics Classification System
DoE	Design of Experiments
